# Eye Contact Perception in the West and East: A Cross-Cultural Study

**DOI:** 10.1371/journal.pone.0118094

**Published:** 2015-02-25

**Authors:** Shota Uono, Jari K. Hietanen

**Affiliations:** 1 Faculty of Human Health Sciences, Graduate School of Medicine, Kyoto University, Kyoto, Japan; 2 Human Information Processing Laboratory, School of Social Sciences and Humanities, University of Tampere, Tampere, Finland; Harvard Medical School, UNITED STATES

## Abstract

This study investigated whether eye contact perception differs in people with different cultural backgrounds. Finnish (European) and Japanese (East Asian) participants were asked to determine whether Finnish and Japanese neutral faces with various gaze directions were looking at them. Further, participants rated the face stimuli for emotion and other affect-related dimensions. The results indicated that Finnish viewers had a smaller bias toward judging slightly averted gazes as directed at them when judging Finnish rather than Japanese faces, while the bias of Japanese viewers did not differ between faces from their own and other cultural backgrounds. This may be explained by Westerners experiencing more eye contact in their daily life leading to larger visual experience of gaze perception generally, and to more accurate perception of eye contact with people from their own cultural background particularly. The results also revealed cultural differences in the perception of emotion from neutral faces that could also contribute to the bias in eye contact perception.

## Introduction

The eyes have a universal language. Humans use information from gaze direction to understand others’ attentional focus and mental state [1] and to maintain social relationships with others [2]. It has been proposed that the structure of the human eye evolved under the pressure of the need for coordinated behavior with others [3]. In contrast to the eyes of other primates, the human eye has a distinctive structure consisting of a white sclera and dark iris. This allows the discrimination of direct and averted gaze and easy recognition of others’ attentional focus.

The function of direct gaze and its impact on other social cognitive functions throughout human development have been extensively investigated [4]. Experimental studies have indicated that a face with a direct gaze rapidly attracts our attention [5]. Human infants prefer faces with a direct gaze over those with an averted gaze since birth [6]. Previous studies have also demonstrated that observing the direct gaze of others elicits higher skin conductance responses [7], enhanced heart rate deceleration responses [8], greater visual event-related brain potentials [9,10], and greater left-lateralized frontal EEG activity [11]—a pattern of EEG activity associated with approach motivation—than observing an averted gaze. Furthermore, it has been shown that a direct gaze enhances various social cognitive functions such as face memory [12], joint attention [13], and empathy [14]. These findings suggest that the detection of a direct gaze has great significance for human social interaction.

The detection of a self-directed gaze is often the starting point for social interaction, and eye contact plays a crucial role in regulating face-to-face interaction. Cultural differences in eye contact perception appear to be a relevant topic for research because of the increase in mobility and social interaction among people of different cultural backgrounds. However, it currently remains unknown whether eye contact perception differs among people with different cultural backgrounds. Previous studies have investigated how accurately people, in general, can discriminate another individual’s gaze direction. These studies have shown that humans can quite accurately discern where a person is looking [15,16]. Consistent with the importance of eye contact in social interaction, other studies have demonstrated that observers can discriminate gaze direction more accurately when the stimulus face is directly looking at them than when the gaze is directed toward other directions [17]. However, when observers were asked to judge whether they felt another person’s gaze was directed at them, they accepted considerable deviations from the “true” (direct) eye contact as a direct gaze [18–21].

There are well-motivated reasons for expecting the bias in detecting a self-directed gaze to vary among people of different cultural backgrounds. First, although a direct gaze universally serves important social functions, attention to faces with a direct gaze differs across cultures. Studies using eye-tracking methodology have demonstrated that East Asians look at the center of a face, while Westerners alternate their focus along a triangle formed by the eyes and mouth when they are required to learn and recognize facial identity [22,23]. However, when recognizing facial expressions Japanese participants attend to the eyes, while Americans focus on the mouth [24,25]. These studies also suggest that the cultural differences in attention to faces with a direct gaze are task dependent.

Second, eye contact behavior differs among cultures. Maintaining eye contact during social interaction is a more important principle for Western Europeans than for East Asians [26]. While maintaining eye contact is positively evaluated by Western Europeans, it is not the case with people of East Asian cultural backgrounds [27]. In fact, in Japanese culture, people are taught not to maintain eye contact with others because too much eye contact is often considered disrespectful. For example, Japanese children are taught to look at others’ necks because this way, the others’ eyes still fall into their peripheral vision [28]. Consistent with this, previous studies have demonstrated, for example, that the Japanese show less eye contact than Canadians during face-to-face interaction [29,30]. These findings suggest that Western Europeans may be more motivated to search for and detect others’ direct gaze during social interaction, and because of their considerable visual experience in perceiving eye contact, they might be less biased in considering slightly averted gazes to be self-directed. This may hold particularly true for faces from their own cultural background relative to faces from other cultural background (with whom they have less visual experience).

Third, it is possible that cultural differences in discerning information about others emotions might also exert an effect on eye contact perception, even when the face in question does not clearly express any emotion. It has been observed from previous research that the perception of gaze direction is modulated by factors not related to gaze direction. For example, observers are more likely to perceive an averted gaze as direct when the face stimulus expresses a happy or angry emotion than when it shows a neutral expression [31–34]. Another line of research has indicated that observers with high levels of social anxiety tend to perceive averted gazes as direct [19], especially when such gazes appear on angry faces [35]. These findings suggest that the emotional expression on the observed face, as well as the observer’s subjective evaluation of it, modulates eye contact perception. Previous studies have shown cultural differences in the emotional intensity ratings of others’ facial expressions. Some studies suggest that the Japanese infer subjective emotions of others to be stronger relative to the Westerners perception. Japanese observers have been shown to perceive subjective emotions on a model displaying facial emotions as more intense than North American observers [36]. Another study also asked Japanese and American participants to rate the intensity of a model’s facial expressions and their perceptions of the model’s subjective experiences [37]. When the model expressed emotion at a low intensity level, Japanese participants gave higher intensity ratings to their perceptions of the model’s subjective experience than to the model’s external display. A recent study investigated cultural differences in autonomic responses and evaluative ratings when participants observed direct and averted gazes of same-culture individuals displaying neutral expressions [8]. They found that Japanese participants rated a face with a direct gaze as angrier, less approachable, and marginally less pleasant than Finnish participants. Further, faces with a direct gaze were rated as sadder than those with an averted gaze by Japanese but not Finnish participants. However, it remains unknown whether Japanese and Finnish participants read emotions differently in neutral faces from their own and other cultures.

This study investigated the cultural differences in eye contact perception among Finnish (European) and Japanese (East Asian) individuals. We presented Finnish and Japanese faces with neutral expressions and various gaze directions (2°, 4°, 6°, 8°, 10° to the left and right, and 0°) to the participants. Finnish and Japanese participants were asked to determine whether the stimulus face was looking at them. People from Western cultures show more eye contact than those from Japan [29,30]. Thus, as Western Europeans have considerable experience processing gazes directed at them in faces from their own cultures, we hypothesized that Finnish participants should be less biased than Japanese participants in considering slightly averted gazes to be directed at them. Moreover, we predicted that Finnish participants would be less biased in considering slightly averted gazes from Finnish faces to be directed at them than gazes from Japanese faces. On the other hand, we expected that the responses of the Japanese participants would not differ between faces coming from their own and others’ cultures. To investigate the effect of cultural differences on the interpretation of others’ emotions, participants were asked to rate face stimuli in terms of emotion (anger, disgust, fear, neutrality, happiness, sadness, and surprise) and other affect-related dimensions (pleasantness, arousal, dominance, and warmth) after the gaze direction judgment task. Finally, participants were also asked to complete questionnaires investigating their degree of autistic traits and social phobia.

## Material and Methods

### Ethics statement

The experiment was conducted in accordance with the Declaration of Helsinki. In accordance with Finnish regulations (Act on Medical Research and Decree on Medical Research 1999, amended 2010), specific ethics approval was not necessary for this kind of study in Finland. We also did not obtain specific ethics approval for this research in Japan, but the experimental procedure was approved as a part of another study by the local ethics committee of Kyoto University Graduate School and Faculty of Medicine. Written informed consent was obtained from all participants.

### Participants

Participants in this study included 30 Finnish (five males) and 30 Japanese (six males) young adults. The Finnish and Japanese participants were recruited from the student populations of the University of Tampere in Finland and Kyoto University in Japan, respectively. All participants were above 18 years (Finnish: mean ± *SD* = 22.97 ± 4.60; Japanese: mean ± *SD* = 21.80 ± 3.37), and there was no difference in the chronological age between the two groups (independent *t*-test, *t* (58) = 1.12, *p* > .10).

### Stimuli

Face photographs of eight Japanese (four females) and eight Finnish models (four females) were taken. The models sat on a chair and rested their heads against a wall behind them to stabilize their head orientation. A camera was located in front of the models, approximately 110 cm away from their heads. The center of the lens was at eye level, between the models’ eyes. To help create photographs with an averted gaze, a horizontal bar was positioned behind the camera, at the eye level, approximately 155 cm away from the models’ heads. Fixation points were attached on the bar at 5.4, 10.8, 16.3, 21.8, and 27.3 cm to right and left of the 0 point, the midline. These fixation points corresponded to a direct gaze and averted gaze of 2°, 4°, 6°, 8°, and 10° to the left and right. During shooting, the models were asked to fixate on each of the markers in turn, alternating in increasing angles from side to side. In the starting 0° gaze condition, the models were asked to fixate on the center of the camera lens because the camera occluded the 0° point on the bar. The models were asked to keep their faces neutral and to change their gaze direction without making any other movement. During shooting, the photographer carefully monitored the models’ possible head movements using markers for the eyes and chin position on a camera monitor.

**Fig 1:**
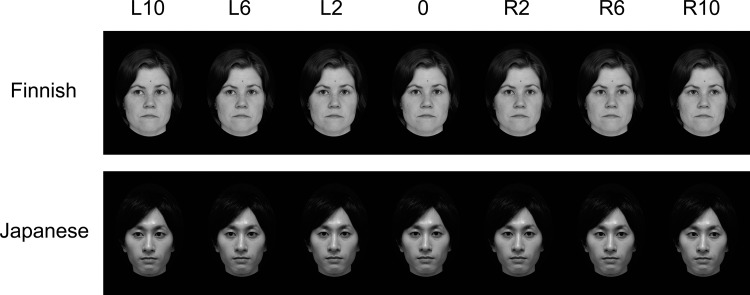
Examples of Finnish and Japanese stimulus faces with various gaze directions. The figure illustrates straight gaze (0°) and gazes averted at 2°, 6°, and 10° to the left and right. Although not illustrated, note that the experiment also included gazes averted at 4° and 8° to the left and right.

After a couple of sets of photographs, the best set (2°, 4°, 6°, 8°, 10° to the left and right, and 0°) was selected for each model. These images were changed to grayscale and were cropped in an ellipse 10.2° wide and 13.8° high using Photoshop (Adobe). Furthermore, we removed the reflections of lighting in the irises from all the images. Examples of the stimuli are shown in Fig. 1.

The photograph sessions for the Finnish and Japanese models were conducted in two laboratories in Tampere and Kyoto, respectively, by the same photographer, the first author (S.U.). Although we carefully followed the same procedure in both laboratories, we wanted to confirm that there would be no differences in gaze direction between the Finnish and Japanese faces and that the respective gaze deviations to the left and right would be of the same size. The distance between the center of the iris and the outer corner of an eye was measured for both eyes in each image. We calculated the average gaze deviation of both eyes at each gaze angle (See Table 1) and subjected these measures to 2 (cultural background of the stimulus face: Finnish and Japanese) × 2 (gaze direction: right and left) between-subject design ANOVAs run separately for each gaze angle (2°, 4°, 6°, 8°, and 10°). ANOVAs revealed no significant main effects or interactions for any gaze angle (all *F*s (1, 28) < 1.01, *p*s > 0.32). This shows that the degrees of gaze aversion at each gaze angle did not differ with respect to the cultural background of the face and gaze direction.

**Table 1:** The average gaze deviation of both eyes at each gaze angle.

(a) Average (with SD) gaze deviation (in pixels) of the left and right eyes in the left-averted gaze conditions					
Stimulus Faces	L2	L4	L6	L8	L10
Finnish	0.80(0.18)	1.60(0.28)	2.43(0.35)	3.33(0.44)	4.17(0.45)
Japanese	0.86(0.19)	1.64(0.15)	2.48(0.33)	3.26(0.46)	4.11(0.46)
(b) Average (with SD) gaze deviation (in pixels) of the left and right eyes in the right-averted gaze conditions					
Stimulus Faces	R2	R4	R6	R8	R10
Finnish	0.88(0.23)	1.74(0.12)	2.54(0.23)	3.28(0.24)	4.22(0.30)
Japanese	0.84(0.18)	1.65(0.26)	2.45(0.32)	3.28(0.40)	4.18(0.46)

### Design

The gaze direction judgment task was constructed as a three-factor design with the cultural background of the participant (Finnish and Japanese) as an independent-measures factor, and the cultural background of the face stimuli (Finnish and Japanese) and gaze angle (0°, 2°, 4°, 6°, 8°, and 10°) as repeated-measures factors.

### Apparatus

Stimulus presentation and data acquisition were controlled by presentation software (Neurobehavioral System) running on a Windows computer (Microsoft). The stimuli were presented on 17-inch CRT monitors (screen resolution: 1024 × 768 pixels; refresh rate: 75 Hz).

### Procedure

Gaze direction judgment task

**Fig 2:**
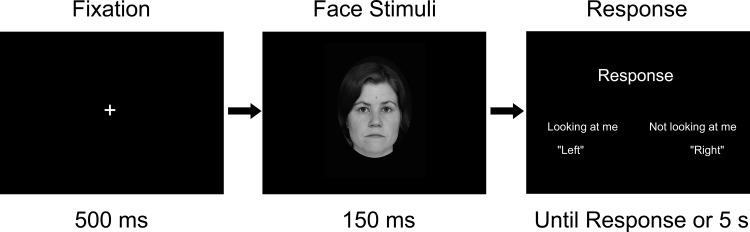
Sequence of events in a single trial.

The sequence of events for a single stimulus presentation trial is shown in Fig. 2. At each trial, a fixation cross was first presented at the center of the screen for 500 ms. Then, a Finnish or a Japanese face with a direct or averted gaze was presented. After 150 ms, the face disappeared and the response window appeared on the screen. Participants were asked to answer whether the face was “looking at me” or “not looking at me” as accurately as possible. At each trial, the response window gave instructions on the use of assigned buttons (right and left button of a mouse) for each response. The instruction remained on the screen until a response was given. If 5000 ms elapsed with no response, the next trial was started.

Each stimulus was presented once for each participant (16 models × 11 gaze directions); thus, the task consisted of 176 trials, presented in two separate blocks. The trials were presented in random order, and the order of the assigned buttons for each response was counterbalanced across the participants. Participants were allowed to rest between the blocks. To familiarize participants with the task procedure, five practice trials preceded the experimental trials.

The rating task

After the completion of the gaze judgment task, participants evaluated the stimuli (Japanese and Finnish faces with direct and averted gaze) using the following scales in three separate blocks. In the first block, participants were asked to look at the stimuli and rate their subjective pleasantness (1 = very unpleasant; 9 = very pleasant), and arousal (1 = very calm; 9 = very aroused) using a 9-point Likert scale. For faces with an averted gaze, only the 10° gaze aversion was shown. Half of the faces (both Japanese and Finnish) had their gaze averted to the left, while the other half had it averted to the right. In the second block, the same stimuli were shown again, and now the task was to evaluate how dominant (1 = submissive, 9 = dominant) and warm (1 = cold, 9 = warm) the person in the picture appeared. In the third block, only faces with a direct gaze were shown, and the participants assessed how intensely the faces reflected each of the following emotions: anger, disgust, fear, neutrality, happiness, sadness, and surprise (1 = not reflecting at all; 9 = reflecting too much). Within each block, a given face remained on the screen while it was being rated along each scale in turn. To avoid any confusion, the scales presented below the stimuli were always named. The order of the blocks was the same for all participants, and the order of the trials was randomized in each block. There was no time limit for the ratings. A face and the name of the scale remained on the screen until a response was made.

Questionnaires

Participants filled out the Autism Spectrum Quotient (AQ) and the Social Phobia Scale (SPS) after completing the experiment. The Japanese version of the AQ [38] and the SPS [39] were used for Japanese participants. For Finnish participants, these two scales were translated into Finnish. The scores in the AQ (Finnish: mean ± *SD* 12.90 ± 5.67; Japanese: mean ± *SD* = 21.70 ± 6.24) and the SPS (Finnish: mean ± *SD* = 17.80 ± 8.10; Japanese: mean ± *SD* = 27.30 ± 12.78) were significantly higher for Japanese than for Finnish participants (AQ: *t* (58) = 5.71, *p* < .001; SPS: *t* (58) = 3.43, *p* = .001).

### Data analysis

Trials with no response and those with response times shorter than 150 ms after stimulus presentation were excluded from analyses. Following previous studies [32, 33], the results were analyzed from data collapsed across the left and right gaze directions. For the gaze direction judgment task, the participants’ percentages of “looking at me” responses in each condition were subjected to a 2 (cultural background of the participant) × 2 (cultural background of the stimulus face) × 6 (gaze angle) mixed-design ANOVA.

Furthermore, we calculated the point of subjective equality (PSE) [40], a gaze deviation degree with a 50% probability of eye-contact acceptance for each participant using a binary logistic regression model [31, 34]. These values were subjected to a 2 (cultural background of the participant) × 2 (cultural background of the stimulus face) mixed-design ANOVA.

For the rating tasks in the first two blocks (pleasantness, arousal, dominance, and warmth), average scores in each condition were first calculated for each participant, and these average scores were then analyzed using a 2 (cultural background of the participant) × 2 (cultural background of the stimulus face) × 2 (gaze direction) mixed-design ANOVA. For the emotion rating task, each participant’s average scores were analyzed using a 2 (cultural background of the participant) × 2 (cultural background of the stimulus face) × 7 (emotion) mixed-design ANOVA. Significant interactions were followed up with simple effects analyses.

When the sphericity assumption was violated, probability values were evaluated with Greenhouse–Geisser adjustments for degrees of freedom. In the preliminary analysis, the scores of the AQ and SPS questionnaires were entered into an ANOVA of gaze direction judgment as covariates. However, the results showed that there were no significant interactions between these scores and the other factors (*p* > .10). Thus, we will not report the effects of the AQ and SPS scores in the results section.

## Results

### Gaze direction judgment task

**Fig 3:**
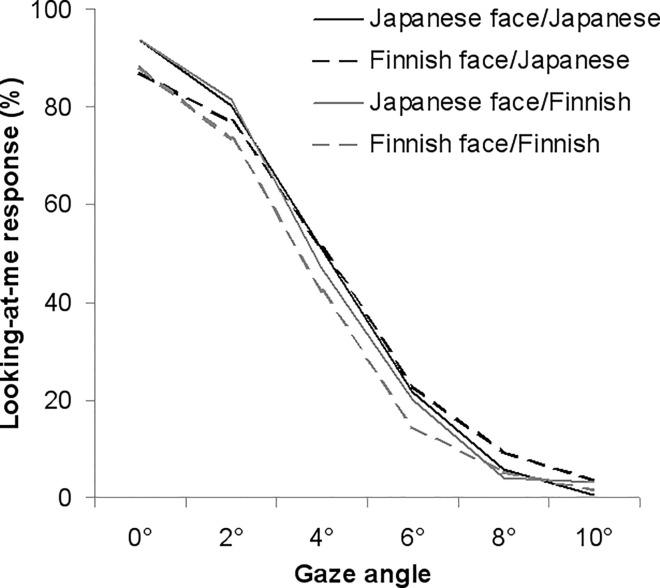
Means of the percentage of looking-at-me responses. Looking-at-me responses are indicated as a function of gaze direction for Finnish and Japanese faces of Finnish and Japanese participants.

Looking-at-me responses were subjected to participants’ cultural background × stimulus faces’ cultural background × gaze direction ANOVA. The main effects of gaze direction (*F* (5, 290) = 1079.74, *p* < .001) and cultural background of the stimulus face (*F* (1, 58) = 8.12, *p* = .006) were observed (see Fig. 3). Predictably, the proportion of looking-at-me responses decreased with increasing gaze angles away from the direct gaze. Overall, the proportion of looking-at-me responses was higher for Japanese than Finnish faces. Essentially, there was a significant interaction between participants’ and stimulus faces’ cultural backgrounds (*F* (1, 58) = 5.86, *p* = .019). A follow-up analysis revealed that, overall, Finnish participants gave more looking-at-me responses to Japanese than Finnish faces (*F* (1, 29) = 12.30, *p* = .002), while the cultural background of stimulus faces had no effect on Japanese participants’ responses (*F* (1, 29) = 0.11, *p* = .748).

A significant interaction between gaze direction × stimulus face’s cultural background was also found (*F* (5, 290) = 5.02, *p* = .001). A follow-up analysis showed that Japanese faces induced more looking-at-me responses than Finnish faces in the 0° and 2° gaze conditions (0°: *F* (1, 58) = 8.83, *p* = .004; 2°: *F* (1, 58) = 9.11, *p* = .004). In contrast, Finnish faces induced more looking-at-me responses than Japanese faces in the 8° gaze condition (*F* (1, 58) = 4.36, *p* = .040).

**Fig 4:**
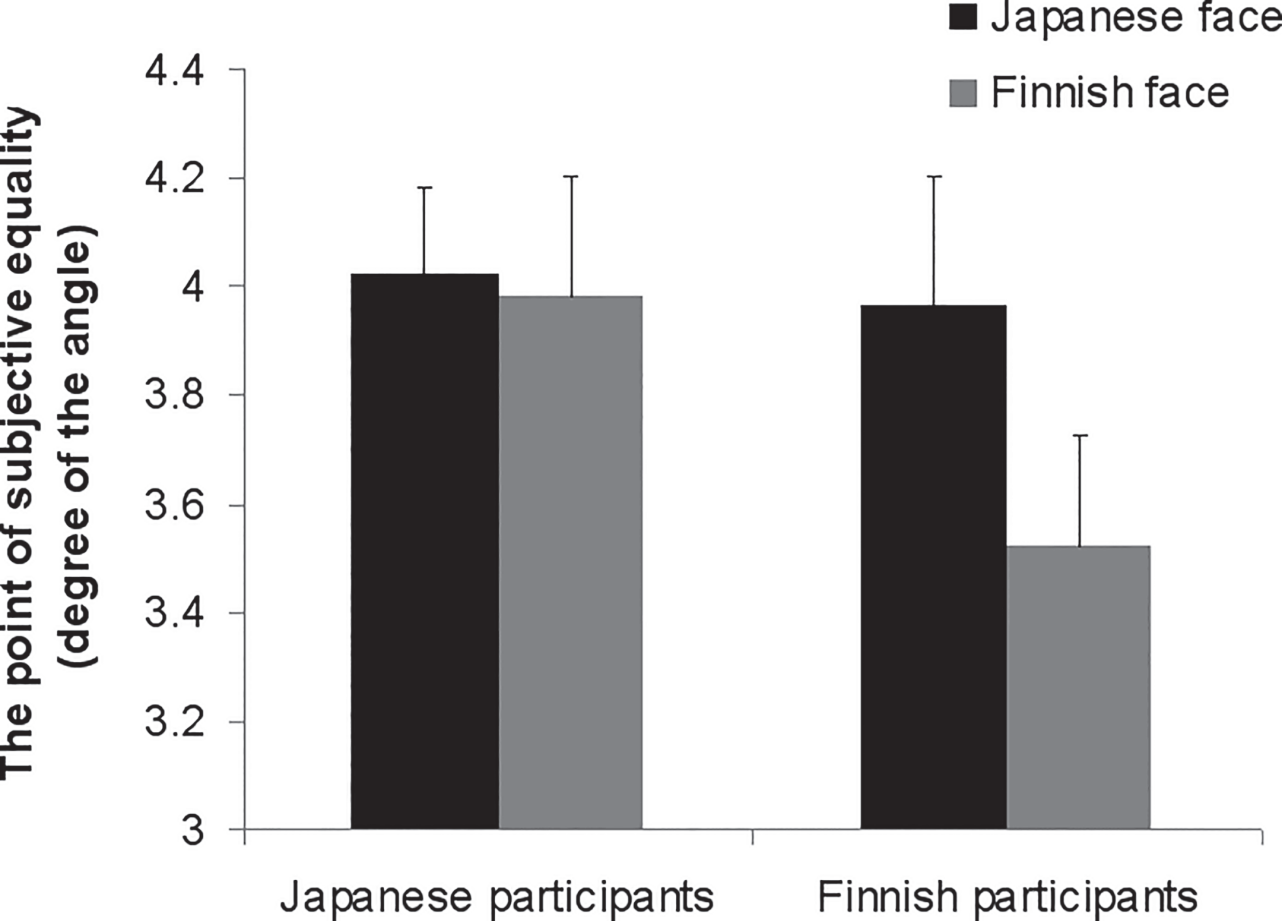
The point of subjective equality between self-directed and averted gaze.

An ANOVA on the PSE values revealed the same pattern of results described above (see Fig. 4). We found a main effect of stimulus faces’ cultural background (*F* (1, 58) = 4.99, *p* = .029), indicating that the gaze deviation degree with a 50% probability of eye-contact acceptance was greater for Japanese than Finnish faces. There was also a marginally significant interaction between participants’ and stimulus faces’ cultural backgrounds (*F* (1, 58) = 3.45, *p* = .068), indicating that gaze deviation degree with 50% probability of eye-contact acceptance in Finnish participants was greater for Japanese than Finnish faces (*F* (1, 29) = 7.13, *p* = .012). In contrast, stimulus faces’ cultural backgrounds had no effect on Japanese participants’ results (*F* (1, 29) = 0.09, *p* = .770).

### Rating tasks

Subjective pleasantness and arousal

The results for the ratings of subjective pleasantness and arousal are shown in Table 2. For the pleasantness ratings, ANOVA revealed a significant main effect of participants’ cultural background (*F* (1, 58) = 4.76, *p* = .033). Finnish participants gave faces higher ratings for pleasantness than Japanese participants. A main effect of gaze direction was also significant (*F* (1, 58) = 4.92, *p* = .031). A direct gaze was rated more pleasant than an averted gaze, regardless of the stimulus faces/participants’ cultural background.

**Table 2:** The results for the ratings of subjective pleasantness and arousal.

(a) Means (with SD) of the pleasantness ratings for different stimulus faces of Finnish and Japanese participants				
	Finnish Faces		Japanese Faces	
Participants	Direct	Averted	Direct	Averted
Finnish	4.80 (0.93)	4.80 (0.94)	4.83 (0.87)	4.69 (0.78)
Japanese	4.48 (0.79)	4.16 (0.83)	4.64 (1.00)	4.24 (0.85)
(b) Means (with SD) of the arousal ratings for different stimulus faces of Finnish and Japanese participants				
	Finnish Faces		Japanese Faces	
Participants	Direct	Averted	Direct	Averted
Finnish	4.39 (1.17)	3.93 (0.90)	4.50 (1.10)	4.10 (1.11)
Japanese	4.75 (1.14)	4.65 (1.11)	4.57 (1.08)	4.29 (0.99)

For the arousal ratings, ANOVA showed a significant main effect of gaze direction (*F* (1, 58) = 8.99, *p* = .004), indicating that a direct gaze was rated as more aroused than an averted gaze. There was also a significant interaction between the cultural background of stimulus faces and that of participants (*F* (1, 58) = 5.52, *p* = .022). A follow-up analysis indicated that Japanese participants tended to rate Finnish faces as more aroused than Japanese faces (*F* (1, 29) = 3.65, *p* = .066). The cultural background of the stimulus faces had no effect on Finnish participants’ arousal ratings (*F* (1, 29) = 1.87, *p* > .10).

Dominance and warmth

The results of the dominance and warmth ratings are shown in Table 3. For the dominance ratings, there was a significant main effect of gaze direction (*F* (1, 58) = 42.21, *p* < .001), indicating that a direct gaze was rated as more dominant than an averted gaze. There was also a significant interaction between participants’ cultural background and gaze direction (*F* (1, 58) = 6.02, *p* = .017). Follow-up analyses revealed no significant simple main effect of participants’ cultural background for a direct gaze (*F* (1, 58) = 1.31, *p* > .10), while Japanese participants rated faces with averted gazes as more dominant than Finnish participants did (*F* (1, 58) = 4.41, *p* = .040). ANOVA also revealed a significant interaction between the cultural backgrounds of the stimulus faces and participants (*F* (1, 58) = 13.48, *p* < .001). A follow-up analysis showed that Finnish participants rated Japanese faces as more dominant than Finnish faces (*F* (1, 29) = 8.74, *p* = .006), while Japanese participants rated Finnish faces as more dominant than Japanese faces (*F* (1, 29) = 5.58, *p* = .025).

**Table 3:** The results of the dominance and warmth ratings.

(a) Means (with SD) of the dominance ratings for different stimulus faces of Finnish and Japanese participants				
	Finnish Faces		Japanese Faces	
Participants	Direct	Averted	Direct	Averted
Finnish	5.46 (0.64)	4.53 (0.79)	5.96 (0.69)	4.89 (0.91)
Japanese	5.75 (0.73)	5.31 (0.67)	5.31 (1.12)	4.84 (1.02)
(b) Means (with SD) of the warmth ratings for different stimulus faces of Finnish and Japanese participants				
	Finnish Faces		Japanese Faces	
Participants	Direct	Averted	Direct	Averted
Finnish	4.69 (0.87)	4.62 (0.89)	4.46 (0.96)	4.62 (0.95)
Japanese	4.12 (0.98)	4.03 (0.74)	4.08 (0.93)	4.08 (1.02)

For the warmth ratings, there was only a significant main effect for participant’s cultural background (*F* (1, 58) = 7.85, *p* = .007), indicating that Finnish participants rated faces as being warmer than did Japanese participants.

Emotion

The results of the emotion ratings are shown in Table 4. Overall, Japanese participants rated neutral faces as more emotional than did Finnish participants (*F* (1, 58) = 16.16, *p* < .001). Importantly, the ANOVA revealed a significant three-way interaction between the cultural backgrounds of participants and stimulus faces and emotion (*F* (6, 348) = 10.93, *p* < .001). Thus, we divided the data and conducted a 2 (stimulus faces’ cultural background) × 7 (emotion) ANOVA for Japanese and Finnish participants separately.

**Table 4:** The results of the emotion ratings.

(a) Mean ratings (with SD) of each emotion for Finnish and Japanese faces in Finnish participants							
Stimulus Faces	Anger	Disgust	Fear	Neutral	Happiness	Sadness	Surprise
Finnish	2.85 (1.09)	2.80 (1.14)	3.20 (1.36)	5.29 (1.68)	1.90 (0.51)	3.25 (1.52)	2.63 (0.86)
Japanese	3.38 (1.09)	3.33 (1.38)	2.35 (0.90)	5.03 (1.38)	1.95 (0.52)	2.57 (1.37)	2.25 (0.47)
(b) Mean ratings (with SD) of each emotion for Finnish and Japanese faces in Japanese participants							
Stimulus Faces	Anger	Disgust	Fear	Neutral	Happiness	Sadness	Surprise
Finnish	4.80 (1.22)	4.51 (1.26)	3.43 (1.39)	4.40 (1.35)	3.15 (0.95)	3.70 (1.43)	3.16 (1.08)
Japanese	4.04 (1.16)	4.32 (1.27)	2.97 (1.30)	5.02 (1.42)	3.04 (1.10)	3.47 (1.06)	2.67 (1.11)

For Japanese participants, a significant main effect was found for stimulus faces’ cultural background (*F* (1, 29) = 15.22, *p* < .001) and emotion (*F* (6, 174) = 19.25, *p* < .001). There was also significant interaction between stimulus faces’ cultural background and emotion (*F* (6, 174) = 8.06, *p* < .001). Finnish faces were rated as more expressive of anger, fear, and surprise than Japanese faces (anger: *F* (1, 29) = 23.99, *p* < .001; fear: *F* (1, 29) = 9.79, *p* = .004; surprise: *F* (1, 29) = 8.92, *p* = .006); while Japanese faces were rated as more neutral than Finnish faces (*F* (1, 29) = 20.19, *p* < .001)

For Finnish participants, there were also significant main effects of emotion (*F* (6, 174) = 32.79, *p* < .001) and stimulus faces’ cultural background (*F* (1, 29) = 6.33, *p* = .018). There was also a significant interaction between emotion and stimulus faces’ cultural background (*F* (6, 174) = 11.21, *p* < .001). The follow-up analysis showed that Finnish faces were rated as more expressive of fear, sadness, and surprise than Japanese faces (fear: *F* (1, 29) = 20.60, *p* < .001; sadness: *F* (1, 29) = 18.74, *p* < .001; surprise: *F* (1, 29) = 9.63, *p* = .004). In contrast, Japanese faces were rated as more expressive of anger and disgust than Finnish faces (anger: *F* (1, 29) = 9.48, *p* = .005; disgust: *F* (1, 29) = 8.43, *p* = .007).

## Discussion

This is the first study to investigate the effects of participants’ and stimulus faces’ cultural background on eye contact perception. Finnish and Japanese participants were asked to judge whether Finnish and Japanese faces were “looking at me” or “not looking at me.” The stimulus faces’ gaze direction was either direct or averted to a varying degree from the direct gaze. The results revealed that the highest frequency of looking-at-me responses was observed for the “true” direct gaze, and there was no abrupt decrease in the looking-at-me responses as the stimulus faces’ gaze direction deviated in 2° increments from 0° to 10°. Previous studies have also shown a similar pattern of looking-at-me responses without an abrupt decrease [20,31–33]. These findings suggest that the perception of eye contact is not categorical but follows a graded function.

There was no overall difference in the percentage of looking-at-me responses between Finnish and Japanese participants. Contrary to our expectation, no evidence was found that Finnish participants were less biased toward considering slightly averted gazes to be directed at them relative to Japanese participants, regardless of the stimulus faces’ cultural background. In the present study, participants’ attention was controlled by fixation to a crosshair, and the stimulus faces were then presented briefly. These task demands might have undermined the differences in eye contact perception for slightly averted gaze between participants coming from different cultural backgrounds.

Interestingly, stimulus faces’ cultural background had an effect on looking-at-me responses among Finnish, not Japanese, participants. Finnish participants gave looking-at-me responses more frequently to Japanese faces than to Finnish faces. Consistent with this, an analysis based on the point of subjective equality revealed that Finnish participants accepted greater deviations from the true eye contact (0°) as directed at them for Japanese faces than for Finnish faces. This suggests that Finnish participants have a smaller bias toward considering a slightly averted gaze as directed at them for Finnish than for Japanese faces. Previous studies have shown that considerable visual experience with specific faces throughout development leads to more effective processing of these faces [41,42]. Finnish participants in this study had seen a larger number of Finnish than Japanese faces during their development; thus, their visual systems are likely to have been trained to discriminate the gaze direction of Finnish faces more accurately. However, the judgments of Japanese participants did not differ between Finnish and Japanese faces. If visual experience plays a role here, as suggested above, why did it not influence the gaze perception of the Japanese participants? We suggest that cultural differences in eye contact behavior might modulate the effect of visual expertise. Although some studies suggested that Japanese participants attend to the eye region when explicitly asked to process facial information [24,25], lengthy eye contact with others is avoided in Japanese culture [27], and the Japanese demonstrate less eye contact than Westerners in daily communication [29,30]. Developing in this cultural context may have restricted Japanese people’s experience in gaze perception to the extent that Japanese participants do not exhibit a same-culture advantage in making gaze direction discriminations between direct gaze and slightly averted gaze directions.

The subjective evaluations of the emotional expressions on stimulus faces provide another way to interpret the effects of the participants’ and stimulus faces’ cultural backgrounds on eye contact perception. It has been proposed that facial signals reflecting the same motivational tendency are combined and therefore processed effectively [43]. Angry or happy expressions, as well as a direct gaze, reflect a desire to approach, while fearful or sad expressions and an averted gaze are signals of a desire for avoidance. Consistent with this, recent studies using the same paradigm as this study have demonstrated that happy and angry expressions reflecting a desire to approach elicit a greater number of looking-at-me responses than neutral and fearful expressions [31–33]. In the present study, Finnish participants rated Japanese faces as expressing more approach-related anger and less avoidance-related fear and sadness than they rated Finnish faces. Thus, Finnish participants’ higher number of looking-at-me responses to Japanese than Finnish faces could have reflected the stronger feelings of approach-related emotions perceived in Japanese faces. In contrast, Japanese participants rated Finnish faces as more intensely expressing both approach-related anger and avoidance-related fear than they rated Japanese faces. Therefore, the effects of approach- and avoidance-related emotions might have canceled each other out, which might have led to the absence of a biased eye contact perception for Japanese vs. Finnish faces among Japanese participants.

The results from the emotion-rating task indicated that Japanese participants rated neutral faces as more emotional than Finnish participants. This finding could reflect differences in display rules between the cultures. In general, when feeling negative or positive emotions, the Japanese suppress or neutralize their facial expressions more than Americans [44]. It has been proposed that the suppressive display rule helps East Asians maintain social relationships in a collectivist society [45]. Thus, Japanese participants might have interpreted neutral expressions as suppressed, and thus interpreted all emotions as more intense. These findings also suggest that because the Japanese are likely to perceive emotion even in the absence of any expressed emotion, the Japanese tendency not to hold eye contact with others may be a way of avoiding high arousal and reciprocal emotional interactions.

The pattern of results from the emotion-rating task might be related to differences in Finnish and Japanese facial structures. Caucasian faces generally have larger eyes than Asian faces. Previous studies have demonstrated that the resemblance of a facial appearance to typical facial expressions modulates the evaluation of neutral faces [46, 47]. For example, large eyes enhance the recognition of a fearful expression, while small eyes facilitate that of an angry expression [48]. Consistent with this, both groups of participants gave higher fear and surprise ratings to Finnish than Japanese faces. This might have contributed to the lower frequency of looking-at-me responses to Finnish than Japanese faces, specifically at around the 0° condition. However, for the anger and dominance ratings, both groups of participants gave higher ratings to faces from cultures other than their own. A recent study demonstrated that out-group faces are associated with angry expressions even when the out-group is defined by a minimal cue [49]. Thus, anger perception in neutral faces from another culture might override the effect of facial structures.

It should be noted that this study has some limitations. First, we did not assess attention allocation in the gaze direction judgment task. Previous studies have demonstrated cultural differences in eye movements while viewing faces [22–24], and the pattern of the cultural differences varies according to task (e.g., identity and expression recognition). To investigate whether cultural differences in attention to faces impact the perception of eye contact, it would be useful for future cross-cultural studies to record eye movements as participants determine gaze direction. Second, although we aimed to investigate cultural differences in eye contact perception, one might ask whether the observed results reflect, in fact, responses to out-group vs. in-group individuals rather than differences between Western and Eastern cultures. Further studies are needed to compare eye contact perception between out-group and in-group members coming from the same cultural background (e.g., Russian vs. Finnish; Chinese vs. Japanese).

## Conclusions

In summary, this study found cultural differences in eye contact perception between Finnish and Japanese participants. The result indicated that Finnish participants were more likely to consider a slightly averted gaze as directed at them when viewing Japanese faces than when viewing Finnish faces. The cultural background of the stimulus faces had no effect on the Japanese participants’ judgments. We suggest that because the Finnish (Westerners) demonstrate more eye contact in their daily lives than the Japanese (East Asians), this leads to better eye contact discrimination and specifically so for faces of their own culture. Another factor possibly explaining the observed pattern of results relates to cultural differences in perceived facial emotions. Finnish observers could have been prone to perceive a slightly averted gaze on Finnish faces as not directed at them because Finnish observers perceived faces from their own culture as expressing more avoidance-motivation-related emotions associated with averted gaze. The cultural background of the face had no effect on Japanese observers’ judgments because Japanese participants rated Finnish faces as more intense than Japanese faces in both approach-related anger and avoidance-related fear. The effects of approach- and avoidance-related emotions thus might have canceled each other out. Therefore, it is possible that cultural differences in facial emotion perception also contribute to the biases in eye contact perception.

## Supporting Information

S1 Dataset Datasets of the gaze direction judgment task and the rating tasks. (XLSX)
